# A study on L-threonine and L-serine uptake in *Escherichia coli* K-12

**DOI:** 10.3389/fmicb.2023.1151716

**Published:** 2023-03-21

**Authors:** Andrey A. Khozov, Dmitrii M. Bubnov, Eugeny D. Plisov, Tatiana V. Vybornaya, Tigran V. Yuzbashev, Gennaro Agrimi, Eugenia Messina, Agnessa A. Stepanova, Maxim D. Kudina, Natalia V. Alekseeva, Alexander I. Netrusov, Sergey P. Sineoky

**Affiliations:** ^1^Kurchatov Complex of Genetic Research, NRC “Kurchatov Institute”, Moscow, Russia; ^2^Department of Microbiology, Faculty of Biology, Lomonosov Moscow State University, Moscow, Russia; ^3^Plant Sciences and the Bioeconomy, Rothamsted Research, Harpenden, United Kingdom; ^4^Department of Biosciences, Biotechnologies and Environment, University of Bari, Bari, Italy; ^5^Mendeleev University of Chemical Technology, Moscow, Russia; ^6^Department of Biochemistry, Faculty of Biology, Lomonosov Moscow State University, Moscow, Russia

**Keywords:** *Escherichia coli*, L-serine uptake, L-threonine uptake, transmembrane transport, amino acid transporter

## Abstract

In the current study, we report the identification and characterization of the *yifK* gene product as a novel amino acid carrier in *E. coli* K-12 cells. Both phenotypic and biochemical analyses showed that YifK acts as a permease specific to L-threonine and, to a lesser extent, L-serine. An assay of the effect of uncouplers and composition of the reaction medium on the transport activity indicates that YifK utilizes a proton motive force to energize substrate uptake. To identify the remaining threonine carriers, we screened a genomic library prepared from the *yifK*-mutant strain and found that *brnQ* acts as a multicopy suppressor of the threonine transport defect caused by *yifK* disruption. Our results indicate that BrnQ is directly involved in threonine uptake as a low-affinity but high-flux transporter, which forms the main entry point when the threonine concentration in the external environment reaches a toxic level. By abolishing YifK and BrnQ activity, we unmasked and quantified the threonine transport activity of the LIV-I branched chain amino acid transport system and demonstrated that LIV-I contributes significantly to total threonine uptake. However, this contribution is likely smaller than that of YifK. We also observed the serine transport activity of LIV-I, which was much lower compared with that of the dedicated SdaC carrier, indicating that LIV-I plays a minor role in the serine uptake. Overall, these findings allow us to propose a comprehensive model of the threonine/serine uptake subsystem in *E. coli* cells.

## 1. Introduction

Studying metabolite transport across the bacterial cytoplasmic membrane is key to understanding how cellular metabolic and regulatory networks interact with the external environment and respond to changes in medium composition. Transmembrane transport mechanisms are of great clinical relevance as they provide insights into host-pathogen interactions and can be promising targets for new drugs. Deep rewiring of metabolite fluxes across the cellular membrane *via* manipulating transport systems is also a pivotal metabolic engineering approach for the development of extensively modified bacterial strains capable of producing valuable chemicals. These strains should possess modified transport systems to ensure efficient excretion of a product and prevent its re-uptake (Okamoto et al., 1997; Kruse et al., 2001, 2002; Doroshenko et al., 2007; Park et al., 2007; Mundhada et al., 2016). In this regard, amino acid transport mechanisms have been extensively studied over the past decades (Burkovski and Krämer, 2002; Marin and Krämer, 2007). One such example is the uptake of L-threonine and L-serine by *Escherichia coli* cells. Owing to the structural similarity between L-threonine and L-serine, they generally share the same transport systems. Among these, SstT is considered a major transporter operating as a Na^+^-dependent symporter (Hama et al., 1987; Ogawa et al., 1997; Kruse et al., 2001). The second carrier for both threonine and serine is the H^+^-dependent TdcC system, which is known to be active exclusively under anaerobic conditions in a rich medium (Sumantran et al., 1990; Ogawa et al., 1997). The SdaC protein is the only known carrier that specifically transports serine across the membrane and shows poor specificity toward threonine. Similar to TdcC, SdaC utilizes the proton motive force to energize serine uptake (Hama et al., 1988; Kayahara et al., 1992; Shao et al., 1994). The last transporter suggested to participate in threonine uptake is LIV-I, an ATP-dependent high-affinity transport system specific for branched-chain amino acids and phenylalanine (Koyanagi et al., 2004). However, there is no direct evidence for the involvement of LIV-I in threonine uptake, as no direct measurement of the threonine transport activity has been reported. The involvement was only inferred from the fact that an excess of threonine efficiently inhibits branched chain amino acid uptake through LIV-I as well as their binding to the substrate-binding component of this transport system (Rahmanian et al., 1973; Robbins and Oxender, 1973; Templeton and Savageau, 1974).

Despite multiple known systems, an additional unidentified carrier mediating threonine uptake, with an activity roughly equal to that of SstT, has been proposed (Kruse et al., 2001). This transport activity is inhibited by serine, indicating specificity toward both amino acids. In this study, we report the identification and characterization of this unknown transporter. Its inactivation made the isolation of a mutant strain without any characterized transport systems possible, which in turn allowed us to unmask and quantify the threonine transport activity of branched-chain amino acid transporters. Overall, being complemented by previous studies, this work provides an integrated view of threonine and serine uptake mechanisms in *E. coli* cells.

## 2. Materials and methods

### 2.1. Bacterial strains and plasmids

The bacterial strains used in this study were derived from *E. coli* K-12. The genotypes and relevant characteristics of all strains, as well as the plasmids used, are listed in Tables 1 and 2, respectively. The strains were constructed using a combination of λ Red recombineering (Datsenko and Wanner, 2000; Bubnov et al., 2018) and P1 transduction (Thomason et al., 2007). The pMW-*liv* plasmid was obtained using *in vivo* gap repair cloning (Datta et al., 2006). For this purpose, the plasmid backbone comprising the pSC101 origin and ampicillin resistance marker was amplified using the primers 5′- TGGCGTTAATGGGGAATGCACAGGCAGTGACGACCATTCCGTTCTGGCATTCTCAATGCTCACGCTGTAG-3′ and 5′- TCGCGTTCTTTTCCAGCTGGCGTACACGCTCAGCGGAAACGCCGTAACGGATTTCTTCCAGAATTGCCATGA-3′, with the low-copy number pMW118 vector (Nippon, Japan) as the template. The resulting PCR product was electroporated into the recombineering-proficient strain B2137 (Bubnov et al., 2022) and ampicillin-resistant colonies were isolated. Successful recombination of the linear PCR-generated vector with the chromosome resulted in recircularization of the plasmid and retrieval of the *livJ-panZ-livKHMGF* chromosomal region into the vector.

**Table 1:** Bacterial strains used in this work.

Strains	Genotype	Relevant characteristic							Source
		Threonine biosynthesis	*sstT*	*tdcC*	*yifK*	*liv*	*brnQ*	*sdaC*	
B3996	*supE ilvA^442^ thrC^1010^ rhtA^23^ tdh::Tn5*	*−*	*wt*	*wt*	*wt*	*wt*	*wt*	*wt*	Laboratory collection
B1044	*supE ilvA^442^ rhtA^23^ tdh::*Tn5 *ΔthrBC ∆tdcBCDE ∆sstT*	*−*	*Δ*	*Δ*	*wt*	*wt*	*wt*		Laboratory collection
MG1655	F-λ*^−^ ilvG-rfb-50 rph-1*	*+*	*wt*	*wt*	*wt*	*wt*	*wt*		Laboratory collection
B2137	MG1655 *Δ[araC-araBAD]::*P_H207_*-lacI-*P_A1O3O4_*-ocr-γβexo-*P_L_*-cat-luxCDABE*	*+*	*wt*	*wt*	*wt*	*wt*	*wt*		Laboratory collection
B1426	MG1655 *∆thrBC ∆sstT ∆tdcBCDE::neo*	*−*	*Δ*		*wt*	*wt*	*wt*		This work
B1817	B1426 *∆yifK::aadA*				*Δ*	*wt*	*wt*		
B1818	B1426 *∆brnQ::aadA*				*wt*	*wt*	*Δ*		
B1851	B1426 *∆yifk ∆brnQ*				*Δ*	*wt*	*Δ*		
B1894	B1426 *∆livKHMGF::cat*				*wt*	*Δ*	*wt*		
B1895	B1426 *ΔyifK ΔbrnQ ∆livKHMGF::cat*				*Δ*	*Δ*	*Δ*		
B2288	B1426 *∆yifK::aadA ∆livKHMGF::cat*				*Δ*	*Δ*	*wt*		
B2289	B1426 *∆brnQ::aadA ∆livKHMGF::cat*				*wt*	*Δ*	*Δ*		
B2374	MG1655 *∆sstT ∆tdcBCDE::neo*	*+*			*wt*	*wt*	*wt*		
B2370	B2374 *∆yifk ∆brnQ*				*Δ*	*wt*	*Δ*		
B2394	B2374 *ΔyifK ΔbrnQ ∆livKHMGF*				*Δ*	*Δ*	*Δ*		
B2395	B2374 *∆yifK ∆livKHMGF*				*Δ*	*Δ*	*wt*		
B2396	B2374 *∆brnQ ∆livKHMGF*				*wt*	*Δ*	*Δ*		
B2429	B2374 *ΔyifK ΔbrnQ ∆livKHMGF ΔsdaC::aadA*				*Δ*	*Δ*	*Δ*	*Δ*	
B2430	B2374 *ΔbrnQ ∆livKHMGF ΔsdaC::aadA*				*wt*	*Δ*	*Δ*	*Δ*	
B2458	B2374 *ΔyifK ΔbrnQ ΔsdaC::aadA*				*Δ*	*wt*	*Δ*	*Δ*	

**Table 2:** Plasmids used in this work.

Plasmids	Relevant characteristics	Source
pBR322	Contains the pMB1 origin of replication and the *bla* (Ap^R^), and *tetA* (Tc^R^) markers; medium-copy-number cloning vector	Laboratory collection
pMW118	Contains the pSC101 origin of replication and *bla* (Ap^R^) marker; low-copy-number cloning vector	Laboratory collection
pBR-*brnQ*	A plasmid for overexpression of the *brnQ* transporter	This work
pMW-*liv*	A plasmid for overexpression of the LIV-I transport system; contains the entire *livJ-panZ-livKHMGF* chromosomal region cloned into the pMW118 vector	This work

The pBR-*brnQ* plasmid was recovered from a genomic library of the B1426 strain, based on its ability to enable the growth of the *yifK*-mutant strain on M9 agar supplemented with both threonine and isoleucine. The insert of chromosomal DNA within the pBR-*brnQ* plasmid corresponds to the coordinates 418,838–421,381 of the MG1655 genome (GenBank accession number U00096.3) and comprises the entire *brnQ* gene along with the first 135 amino acid residues (out of 435) of the *proY* open reading frame.

### 2.2. Media and culture conditions

Bacteria were routinely grown in Luria-Bertani (LB) broth (10 g/l tryptone, 5 g/l yeast extract, and 10 g/l sodium chloride) at 37°C with shaking at 220 rpm. Solid LB medium was prepared by adding 20 g/l agar to the LB broth. When necessary, ampicillin, kanamycin, and chloramphenicol were added to the medium at concentrations of 200, 100, and 20 μg/ml, respectively. Either solid or liquid M9 minimal medium (Sambrook et al., 1989) with 0.2% glucose was used for the phenotype and transport assays. The medium was supplemented with L-threonine, L-isoleucine, L-alanyl-L-threonine, L-serine, and L-leucine as appropriate. The concentrations of these additives are indicated in the Results and figure legends.

### 2.3. DNA manipulations

Standard methods were used for chromosomal DNA isolation, restriction enzyme digestion, agarose gel electrophoresis, ligation, and transformation (Sambrook et al., 1989). DNA amplification was accomplished by PCR using DreamTaq (Thermo Fisher Scientific, Vilnius, Lithuania) or KAPA HIFI (Kapa Biosystems, Wilmington, MA, United States) polymerases. Plasmids were isolated and DNA fragments were extracted using the GeneJET Plasmid Miniprep kit and GeneJET Gel Extraction kit (Thermo Fisher Scientific, Vilnius, Lithuania), respectively.

### 2.4. Genomic library preparation and identification of *yifK* multicopy suppressors

A genomic library was prepared using the chromosome of the B1426 strain and the pBR322 plasmid vector. The isolated genomic DNA was partially digested using Bsp143I, followed by gel purification of fragments 1–5 kb in length. The fragments were then ligated with pBR322, which had been treated with BamHI endonuclease and FastAp alkaline phosphatase. The ligation mixture was electroporated into XL1 Blue cells. The cells were resuspended in 200 ml LB medium containing 200 mg/l ampicillin and grown to saturation. The pooled library was then isolated, diluted to 10 ng/μL, and electroporated into the *yifK*-mutant B1817 strain. Transformants were selected on M9 agar supplemented with 200 mg/l ampicillin, 20 mg/l threonine, and 25 mg/l isoleucine. Plates were incubated for 3 days and the resulting colonies were re-purified on the same medium to be used for plasmid isolation, followed by sequencing of the genomic inserts using primers 5′- GGTTGAGGCCGTTGAGCAC-3′ and 5′- ACATTAACCTATAAAAATAGGCG-3′.

### 2.5. Phenotype assay

A single colony of the strain to be tested was picked and inoculated into 5 ml of LB broth with the appropriate antibiotics in a 50-mL test tube and incubated overnight. Next, a 1 ml aliquot of the overnight culture was precipitated by centrifugation for 1 min at 12000 × *g*, washed twice with M9 medium, resuspended in 1 ml of the same medium with 0.2% glucose, and incubated for 2 h at 37°C and 1,000 rpm to ensure exhaustion of the intracellular threonine. The OD_600_ was then determined, and the cell suspension was diluted with M9 medium to obtain an OD_600_ of 0.1. Dilutions from 10^0^ to 10^−5^ were prepared, and 4 μl aliquots were dropped onto M9 agar with 0.2% glucose, supplemented with additives described in the Results and figure legends. The plates were then incubated at 37°C for 2 days.

### 2.6. Transport assay

Uniformly labeled L-[U-^14^C]threonine and L-[U-^14^C]serine were obtained from Moravek Biochemicals (United States). Carbonyl cyanide m-chlorophenyl hydrazone and monensin sodium salt were obtained from Sigma-Aldrich (USA).

To measure L-[U-^14^C]threonine and L-[U-^14^C]serine transport, cells were grown overnight in M9 medium supplemented with 0.2% glucose at 37°C with antibiotics, if needed. The optical density of the overnight culture was measured. Based on the obtained value, the culture was diluted in 20 ml of the same medium to obtain an initial OD_600_ of 0.0625 and grown until the OD_600_ reached 0.5. Plasmid-carrying strains were grown in the presence of 100 mg/l ampicillin, whereas the other strains were incubated in antibiotic-free M9 medium. All subsequent steps were performed on ice. The cells were harvested by centrifugation at 5000 × *g* at 4°C for 5 min in 50 ml polypropylene tubes. The supernatant was discarded, and the cells were washed once with 35 ml of M9 medium. The pellet was resuspended in 1 ml of M9 medium and transferred to a precooled 1.5 ml tube. The tube was then spun for 45 s in a cooled microcentrifuge rotor at 12000 × *g* and the supernatant was thoroughly aspirated. The pellet was resuspended in 800 μl of M9 medium, and the optical density was measured. The suspension was diluted with M9 medium with 0.2% glucose to obtain an OD_600_ of 6.4, and chloramphenicol was added at a final concentration of 50 μg/ml to stop protein synthesis. Both the cell suspension and labeled substrates dissolved in M9 medium with 0.2% glucose were separately preincubated for 20 min at 37°C. To examine the effect of either CCCP or monensin, they were added to the cell suspension 15 min prior to starting the reaction. Uptake was initiated by adding the cell suspension to the substrate solution to achieve an OD_600_ of 2. The reaction mixture was incubated at 37°C. Then, 50 μl samples were periodically collected and immediately filtered through 0.45-μm GVS North America 13-mm nitrocellulose membranes presoaked in M9 medium on a vacuum manifold, followed by washing with 1 ml of the same medium. For measuring the initial uptake velocity, the reaction time was set to 30 s. Membranes were air-dried at 37°C for 18–20 h and radioactivity was measured using 10 ml of GC-106 scintillation liquid (4 g 2,5-diphenyloxazole and 0.1 g 2,2′-(1,4-phenylene)bis(5-phenyl-1,3-oxazole) dissolved in 1 l of toluene) on a Tri-Carb 4,810 TR liquid scintillation analyzer (PerkinElmer, United States). The amount of radioactivity absorbed by the membrane was determined as the control. For each substrate concentration, the reaction mixture without cells was incubated, filtered, washed, and counted identical to the experimental reactions. The measured values were subtracted from those obtained in the appropriate experiment. Transport activity was expressed as nanomoles of a substrate taken up by 1 mg of dry cellular weight (DCW) in 1 min. The DCW value was calculated based on the OD_600_ of a cell suspension. The ratio was calculated as follows: a 10 ml aliquot of the B2374 cell suspension with OD_600_ 6.4 was prepared for the uptake measurement as described above and centrifuged for 10 min in a 50 ml polypropylene tube at 4000 × *g*. The pellet was washed once with 10 ml of deionized water, resuspended in 1 ml of water, and transferred into a pre-weighed 2 ml tube. The tube was spun for 45 s in a microcentrifuge at 12000 × *g*, and the supernatant was thoroughly aspirated. The cell pellet was then dried to a constant weight at 90°C using an Ohaus Moisture Analyzer MB120 (Ohaus, United States). The ratio was 437 mg DCW per liter of a cell suspension with an OD_600_ of 1. The main steps of these experiments are shown in Supplementary Figure S1.

To determine the kinetic parameters, the initial uptake velocity was plotted against the substrate concentration in double-reciprocal coordinates, and the K_m_ and V_max_ values were calculated using the Lineweaver-Burk equation:


$$\frac{1}{V}=\frac{K_{m}}{V_{\text{max}}}\;\frac{1}{S}+\frac{1}{V_{\text{max}}}$$

where *S* is the substrate concentration, *V_max_* is the maximum velocity of the reaction, and *K_m_* is the Michaelis constant.

All comparisons were made using the two-tailed Student’s *t-*test with unequal variances at a significance level of 5%.

## 3. Results

### 3.1. Identification of transporters involved in threonine uptake

**Figure 1:**
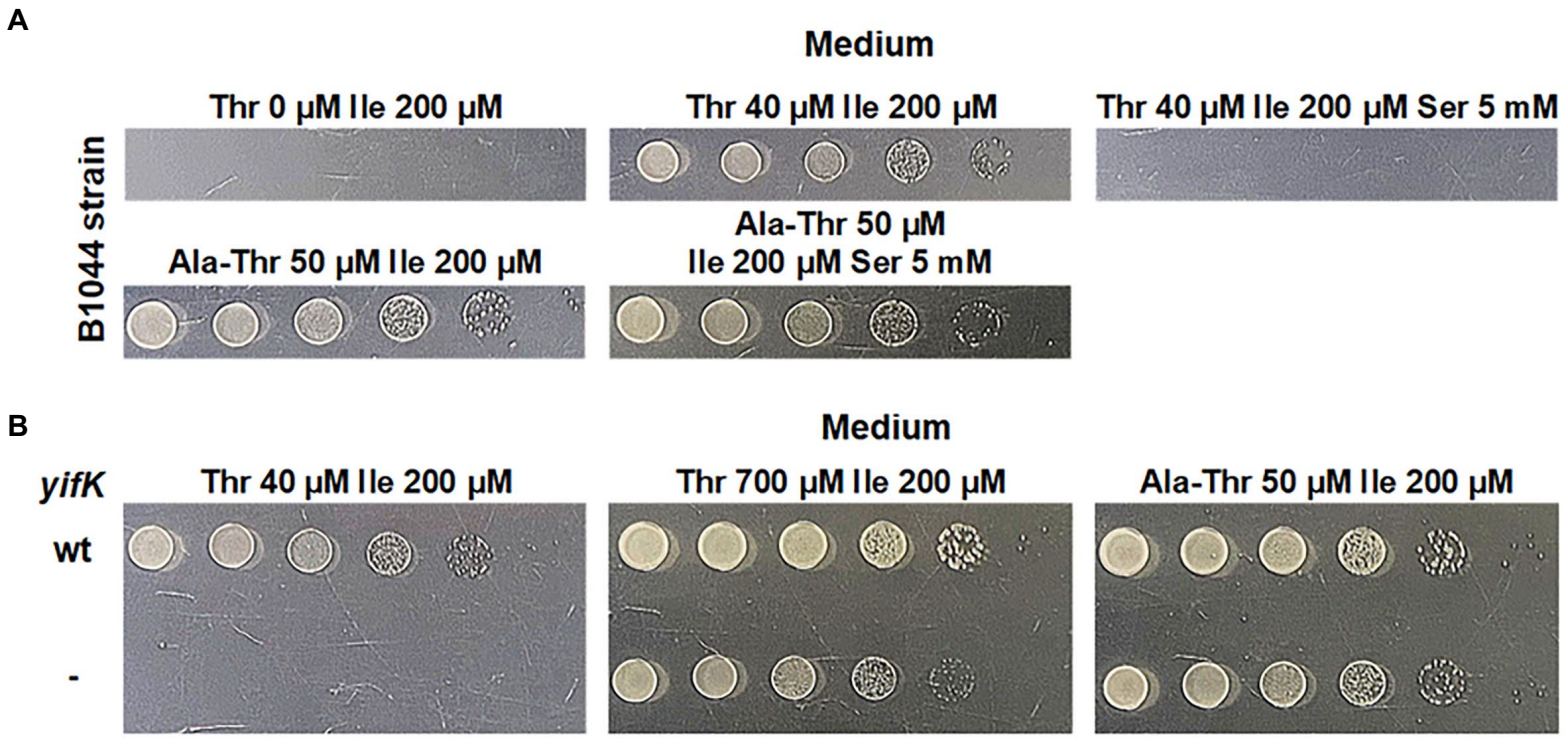
Phenotype assay of the threonine-auxotrophic B1044 strain and its *yifK*^-^ derivative, B1082. The assay was performed using M9 minimal medium with 0.2% glucose and the indicated additives as described under “Phenotype assay” in the Materials and Methods. “Thr,” “Ile,” “Ser,” and “Ala-Thr” stand for L-threonine, L-isoleucine, L-serine, and L-alanyl-L-threonine, respectively. **(A)** Growth assay of B1044 on minimal plates with either L-threonine or L-alanyl-L-threonine as the L-threonine source. L-isoleucine was added to the medium as B1044 is unable to synthesize this amino acid along with L-threonine. L-serine was added to examine whether it inhibits L-threonine uptake. **(B)** Comparison of the phenotypes of B1044 and B1082 strains on minimal plates with either L-threonine or L-alanyl-L-threonine as an L-threonine source.

Our initial rationale for attempting to identify a previously unknown threonine carrier was based on the fact that a strain lacking SstT, the major known threonine and serine transporter, possesses an additional transport system capable of translocating threonine. It was reported to have activity comparable to that of SstT, and to be sensitive to inhibition by an excess of serine (Kruse et al., 2001). We sought to confirm this observation *via* phenotypic analysis. We initiated our experiments with the B3996 strain (Debabov, 2003). Among other mutations (see Table 1 for the genotype), this strain also carried the *ilvA^442^* mutation that confers a deficiency in threonine dehydratase and, consequently, isoleucine auxothrophy. Therefore, the growth medium for this strain and its derivatives was supplemented with isoleucine. Based on B3996, we constructed the threonine auxotroph B1044 by deleting the *thrBC* genes, which encode enzymes catalyzing the last two reactions of the threonine biosynthesis pathway. Further, the known threonine-specific carriers, SstT and TdcC, were inactivated by introducing the corresponding deletions. For semi-quantitative analysis of threonine uptake, we exploited the dependence of the resulting strain growth in minimal medium on the ability of cells to accumulate exogenous threonine. Specifically, we could draw conclusions on the activity of threonine uptake based on a shift in the minimal threshold concentration of threonine in the medium, which allowed growth of the B1044 uptake-deficient auxotroph. First, we determined 40 μM as the threshold concentration of threonine. Then, using the described experimental design, we verified that addition of serine at 125-fold excess compared with threonine prevented the growth of the B1044 strain at this threonine concentration. However, when threonine was replaced with 50 μM L-alanyl-L-threonine dipeptide, inhibition was not observed (Figure 1A). Therefore, we concluded that serine likely inhibits threonine uptake *via* an unknown carrier for both threonine and serine.

To identify the gene(s) encoding these transporter(s), we first assumed an amino acid sequence homology with SstT or TdcC. Using these query sequences and the tblastn algorithm (McGinnis and Madden, 2004) we identified five potential target genes, namely *gltP*, *cyuP*, *yqeG*, *yhjV*, and *sdaC* in the genome of the MG1655 strain (GenBank accession number U00096.3). The latter gene is known to be a serine carrier (Hama et al., 1988; Kayahara et al., 1992; Shao et al., 1994) that is incapable of transporting threonine. However, we decided to retain it for further work to ensure the comprehensiveness of the analysis. Considering the number of possible candidates, we assumed that more than one transporter participates in threonine uptake. In this case, we would not observe a phenotypic change in our test system when a single target gene was disrupted from the set. Therefore, we decided to introduce premature stop codons into all five genes using λ Red-mediated oligo recombination (Ellis et al., 2001; Wang et al., 2009; Sawitzke et al., 2011) to simultaneously obtain all possible combinations of mutations. Upon transforming B1044 cells with a mixture of mutagenizing oligonucleotides and replica plating several thousands of colonies, we isolated a single clone designated as B1082, which exhibited a significantly elevated threonine threshold concentration of 700 μM compared with that of the parental strain (40 μM). Simultaneously, the phenotype on M9 agar supplemented with L-alanyl-L-threonine remained unaltered (Figure 1B). Thus, we concluded that the mutant showed impaired threonine uptake. Surprisingly, sequencing of the targeted loci did not reveal any mutation. Notably, for oligo recombineering, we used a helper plasmid that expresses a dominant negative allele of *mutS* along with the Red operon, whose product transiently inhibits the activity of the methyl-directed mismatch repair system (Bubnov et al., 2018). Thus, the selected mutant may have a spontaneous mutation elsewhere in the genome. Indeed, genome re-sequencing revealed four mutations relative to the parental strain, namely replacements C➔T, C➔T, and A➔T at coordinates 127,840, 1,123,865, and 3,691,123, respectively, and a single nucleotide deletion at coordinate 3,980,951 (all coordinates are in accordance with the GenBank entry U00096.3). Among these, only the last mutation affected the transport protein, as it was a frameshift in the 22nd leucine codon of the *yifK* ORF, which encodes a putative transporter belonging to the Amino Acid-Polyamine-Organocation Superfamily (Saier et al., 2014).

**Figure 2:**
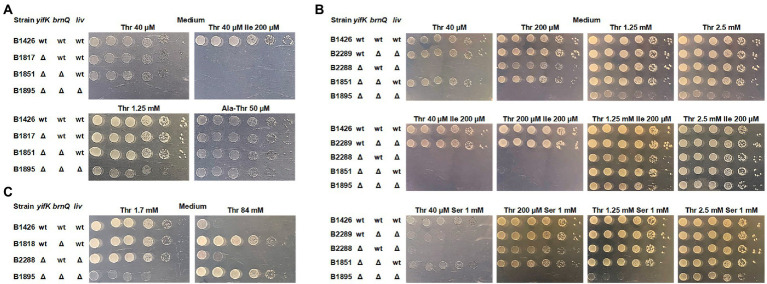
Phenotype assay of the threonine-auxotrophic B1426 strain and its derivatives carrying deletions of genes controlling threonine uptake. The assay was performed using M9 minimal medium with 0.2% glucose and the indicated additives as described under “Phenotype assay” in the Materials and Methods. “Thr,” “Ile,” “Ser,” and “Ala-Thr” abbreviations stand for L-threonine, L-isoleucine, L-serine, and L-alanyl-L-threonine, respectively. **(A)** Growth assay of the B1426 strain and its single, double, and triple mutant derivatives lacking the YifK, BrnQ, and LIV-I systems on minimal plates with either L-threonine or L-alanyl-L-threonine as the L-threonine source. **(B)** Growth assay of the B1426 strain and its double mutant derivatives possessing YifK, BrnQ, and LIV-I as the sole threonine transport system at gradually increasing L-threonine concentrations. L-isoleucine and L-serine were added as substrates competing with L-threonine for transport to examine whether they exhibit an inhibitory effect on the growth of the tested strains. **(C)** Phenotype assay showing the causality of BrnQ in L-threonine toxicity.

For further analysis, we switched to derivatives of the MG1655 strain, as B3996 has a heavily mutagenized genetic background. We re-introduced the same mutations, namely *ΔthrBC ΔsstT ΔtdcBCDE*, thereby producing the B1426 strain, and constructed the isogenic B1817 strain carrying the *ΔyifK* mutation. Intriguingly, we did not observe the expected shift in the threshold threonine concentration for B1817 compared to that for B1426, unless the M9 agar was supplemented with isoleucine (Figure 2A). Notably, none of these two strains required isoleucine for their growth, whereas B1044 required isoleucine because of the *ilvA^442^* mutation. This led us to assume that some isoleucine-specific transport systems contribute to threonine uptake and become unmasked when isoleucine is omitted from the medium. Alternatively, externally added isoleucine can reduce the activity of this putative transporter *via* transcriptional repression. A branched-chain amino acid transporter, LIV-I, has been indirectly associated with threonine transport, as this amino acid could inhibit leucine uptake catalyzed by this carrier (Rahmanian et al., 1973; Robbins and Oxender, 1973; Templeton and Savageau, 1974). Considering that this finding was based on indirect evidence, we aimed to identify the transporter still active in the B1817 strain; to this end, we prepared a genomic library by cloning the chromosomal DNA of this strain in the pBR322 vector to search for genes that could serve as multicopy suppressors of the *yifK* mutation and enable the growth of B1817 on M9 agar supplemented with both threonine and isoleucine. We assumed that amplification of such a gene on a multicopy plasmid could result in increased expression and higher activity of the corresponding carrier, which could be sufficient to provide an adequate rate of threonine uptake even in the presence of isoleucine-mediated inhibition or repression. Indeed, upon transformation of the B1817 strain with a genomic library, we isolated transformants that could grow on minimal medium containing both amino acids. Among these, the most frequent inserts were variable chromosomal regions comprising the *brnQ* gene, which was previously reported to encode the second transport system for branched chain amino acids known as LIV-II (Guardiola and Iaccarino, 1971; Anderson et al., 1976; Antonucci and Oxender, 1986; Ohnishi et al., 1988). Upon introducing the *brnQ* deletion into the B1817 strain, we did not observe any phenotypic changes, including growth inhibition by isoleucine (Figure 2A). The most likely explanation for this result is the isoleucine-inhibitable threonine transport activity of the LIV-I system. Phenotypic analysis of the triple Δ*yifk ΔbrnQ ΔlivKHMGF* mutant B1895 strain revealed that this strain was unable to grow on M9 medium with a threonine concentration lower than 1.3 mM (Figure 2A).

We thus sought to evaluate the contribution of each identified carrier to total threonine uptake. We constructed three double-mutant derivatives of B1426, each of which retained a single transporter. We found that YifK and LIV-I contributed equally, as the corresponding mutants grew normally on minimal agar with the lowest threonine concentration of 40 μM (Figure 2B). Although we were unable to observe any difference between the *ΔyifK* and *ΔyifK ΔbrnQ* mutant strains in the previous experiment, the effect of *brnQ* presence was clearly observable when the B2288 strain retaining this gene was compared to the triple mutant B1895 strain, indicating the relatively small but significant contribution of this carrier (Figure 2B). Intriguingly, we found that the *ΔbrnQ* mutation renders cells threonine-resistant, as the strain B1818 carrying a single *brnQ* mutation (in addition to the *ΔthrBC, ΔsstT,* and *ΔtdcBCDE* mutations), as well as the triple mutant B1895 strain grew on M9 agar with 84 mM threonine, whereas strains with intact *brnQ* did not (Figure 2C). These observations indicate that BrnQ contributes to threonine uptake at significantly higher substrate concentrations than YifK and LIV-I.

Using these three double-mutant strains, we analyzed growth inhibition by serine and isoleucine. As expected, the growth of strain B2289, in which YifK served as a single threonine carrier, was inhibited by an excess of serine, thereby confirming the dual substrate preference of this protein. The B2288 and B1851 strains possessing either BrnQ or LIV-I transport systems did not grow when M9 agar was supplemented with isoleucine. These findings clearly explain the phenotype of the B1044 strain on isoleucine-supplemented agar and the lack of growth defects in the B1817 strain on agar without isoleucine.

### 3.2. Assaying threonine uptake in the mutant strains

Using an *in vitro* transport assay, we sought to verify our conclusions regarding the contribution of the identified transport systems, based on phenotypic analyzes. Specifically, we compared the threonine transport activity of the parental B2374 strain with that of the triple mutant B2394 strain and three double mutant strains B2396, B2370, and B2395, respectively. Notably, these strains are isogenic derivatives of strains B1426, B2289, B1851, and B2289, with the only difference being that the strains used for the transport assay possessed the wild-type *thrABC* operon. We could cultivate these strains in M9 glucose medium without any supplements, which could affect the expression levels of the carriers.

**Figure 3:**
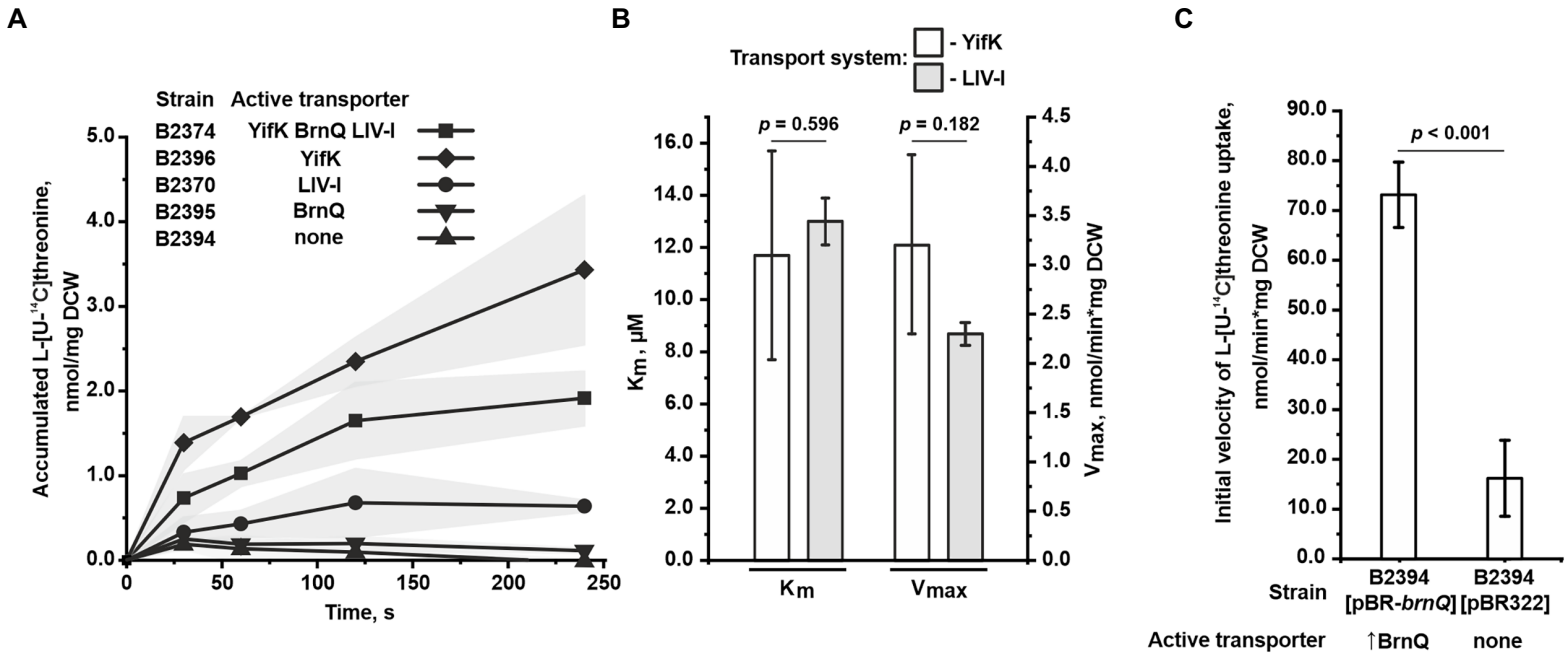
Assay of L-threonine transport activity. The experiments were performed as described under “Transport assay” in the Materials and Methods. The values shown are the average of three biological replicates; the shaded area and error bars indicate SD. The depicted *p-*values were calculated using the two-tailed Student’s *t-*test with unequal variances. **(A)** A time course of L-threonine accumulation by cells of the B2374 strain and its derivatives possessing YifK, BrnQ, or LIV-I as the sole carrier. Measurements were performed at 50 μM L-threonine. **(B)** Evaluation of the kinetic parameters of YifK and LIV-I carriers using L-threonine as a substrate. YifK and LIV-I transport systems were assayed using the B2396 and B2394[pMW-*liv*] strains, respectively. The V_max_ value characteristic of the LIV-I system was adjusted by dividing the value obtained for B2394[pMW-*liv*] cells by factor 9.85 (for more details, see the text under “Assaying the threonine uptake in the mutant strains” in the Results). The values were calculated using Lineweaver-Burk plots obtained with seven concentration points. **(C)** Comparison of L-threonine transport activity in B2394 strain lacking the YifK, BrnQ, and LIV-I carriers with that of B2394[pBR-*brnQ*] overexpressing BrnQ because of the multicopy pBR-*brnQ* plasmid. The vertical arrow in “↑BrnQ” indicates that BrnQ is overexpressed in this strain. The measurements were performed at 800 μM of L-threonine.

In agreement with the phenotypic data, at a threonine concentration of 50 μM, we found that the triple mutant B2394 strain was almost completely unable to accumulate exogenous threonine (Figure 3A). Upon assaying the double-mutant strains, we observed the activity of only two transport systems. Specifically, the B2370 strain, with LIV-I as the sole transport system, accumulated threonine at a significantly lower rate than that of the parental B2374 strain with all three transport systems active. However, threonine uptake was clearly observed in B2370 cells, indicating that threonine is a substrate of the LIV-I system. Intriguingly, the B2396 strain, with YifK as the only carrier, accumulated threonine even faster than the parental B2374 strain. We assume that this inconsistency is possibly attributed to the interplay between transport systems caused by a regulation of expression dependent on the intracellular amino acid pool. However, further studies are required to address this question.

Next, we evaluated the kinetic parameters for YifK and LIV-I using the double mutant strains B2396 and B2370, respectively, in which these transport systems operate as sole threonine carriers. For YifK and threonine as a substrate, the measured K_m_ value was 11.7 ± 4.0 μM, V_max_ was determined to be 3.2 ± 0.9 nmol/min*mg DCW (Figure 3B). When assaying the LIV-I system, we could not obtain data with sufficient quality for fitting with a straight line in the double reciprocal Lineweaver–Burk plot. We considered that this was caused by the relatively significant contribution of some non-specific transport systems in threonine uptake. Therefore, we overexpressed LIV-I to enhance its activity. To do this, we subcloned the entire *livJ-panZ*-*livKHMGF* locus into the low-copy-number pMW118 vector, thereby producing the pMW-*liv* plasmid. Using the triple mutant B2394 strain transformed with pMW-*liv*, we determined the K_m_ and V_max_ of LIV-I for threonine to be 13.0 ± 0.9 μM and 22.2 ± 0.9 nmol/min*mg DCW, respectively (Figure 3B). We realized that this evaluation of the V_max_ value was highly overestimated owing to the overexpression of LIV-I genes. To adjust for this, we measured the ratio of the initial uptake velocities at 100 μM threonine in the B2394[pMW-*liv*] strain and the B2370[pMW118] strain with a single chromosomal copy of the *livJ-panZ*-*livKHMGF* locus, and found it to be 9.85 (data not shown). The V_max_ value of LIV-I expressed from a single copy was then estimated to be approximately 2.3 ± 0.09 nmol/min*mg DCW (Figure 3B).

Despite finding a significant contribution of BrnQ to threonine uptake in the growth experiments, direct assay of transport at 50 μM revealed very little, if any, transport activity mediated by this carrier (Figure 3A). However, the growth data indicated that the phenotype of the wild-type BrnQ became visible at a higher substrate concentration (200 μM) than that of LIV-I and YifK (40 μM), whereas good growth was observed when the threonine concentration reached 1.25 mM. To improve the sensitivity of the transport assay, we overexpressed BrnQ using the triple mutant B2394 strain harboring the multicopy pBR-*brnQ* plasmid retrieved from the genomic library, and measured the uptake activity at 800 μM threonine. We observed an approximately 4.5-fold increase in the transport activity of the strain with the *brnQ-*carrying plasmid above the background level, characteristic of the strain with the empty pBR322 vector (Figure 3C). Thus, several lines of evidence support the direct involvement of BrnQ in threonine uptake. First, the presence of *brnQ* on the multicopy pBR322 plasmid complemented the growth defect of the *yifK-*mutant strain. Second, at growth-limiting threonine concentrations, the wild-type *brnQ* allele conferred a clearly observable growth advantage compared with the triple mutant *ΔyifK ΔbrnQ ΔlivKHMGF* strain. Third, the single *brnQ* mutation (on the *ΔsstT ΔtdcBCDE* background) was sufficient to ensure resistance to a high concentration of threonine. Finally, transport activity was directly detectable when using a strain overexpressing BrnQ. These results indicate that BrnQ constitutes a low-affinity transport system for threonine, which confers a much higher flux than that of YifK and LIV-I. Unfortunately, we could not measure the kinetic parameters of BrnQ, as we did not observe saturation of the uptake rate up to 1 mM of threonine; however, at threonine concentrations higher than 400 μM, the transport activity of the triple mutant strain was significant, which disturbed the results.

### 3.3. YifK is a H^+^-coupled threonine/serine symporter

**Figure 4:**
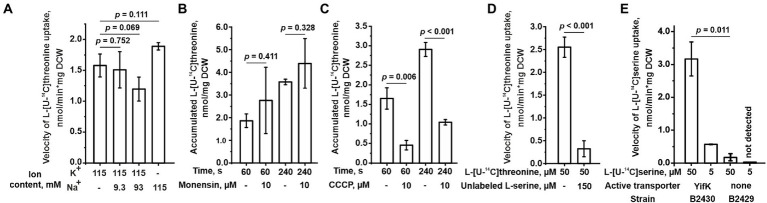
Characterization of the YifK carrier. The experiments were performed as described under “Transport assay” in the Materials and Methods. The values shown are the average of three biological replicates; error bars indicate SD. The depicted *p-*values were calculated using the two-tailed Student’s *t-*test with unequal variances. **(A)** The effect of the ion composition of a reaction mixture on the L-threonine transport activity conferred by YifK. The cells of the B2396 strain possessing YifK as a sole L-threonine carrier were grown in regular M9 medium, but the washing steps and uptake reaction were performed using M9 containing K^+^ or Na^+^ as the sole monovalent cation or a mixture of K^+^ and Na^+^ as indicated under the x-axis. For this, Na^+^-containing salts were replaced with K^+^-containing counterparts and vice versa. To obtain M9 medium with mixtures of K^+^ and Na^+^, the K^+^-based medium was supplemented with an appropriate amount of NaCl. The assay was performed at 50 μM L-threonine. The reaction time was 1 min. **(B)** The effect of monensin on the L-threonine transport activity of YifK. The experiments were performed using cells of the B2396 strain at 50 μM L-threonine. **(C)** The effect of CCCP on the L-threonine transport activity of YifK. The experiments were performed using cells of the B2396 strain at 50 μM L-threonine. **(D)** The effect of excessive unlabeled L-serine on the L-threonine transport activity of YifK. The experiments were performed using cells of the B2396 strain. The reaction time was 1 min. **(E)** The L-serine transport activity conferred by YifK. The experiments were performed using cells of the B2430 strain lacking all known L-serine carriers and its ∆*yifK* derivative B2429 strain at the indicated L-serine concentrations and a reaction time of 1 min.

We sought to identify the mechanism of energetic coupling of transport through YifK. We assumed two of the most common mechanisms in bacterial amino acid transporters, namely, either Na^+^-dependent or H^+^-dependent symport. We measured threonine transport in the B2396 strain and found that replacing Na^+^ with K^+^ in the reaction mixture did not significantly affect the threonine transport activity (Figure 4A). When the reaction mixture containing K^+^ instead of Na^+^ was supplemented with varying concentrations of Na^+^ no positive effect on transport was observed. Furthermore, while assaying threonine uptake through YifK in regular M9 medium, we found that transport was insensitive to 10 μM monensin, a Na^+^/H^+^ exchanger (Figure 4B). Meanwhile, YifK activity was inhibited from 2.8- to 3.7-fold in the presence of 10 μM CCCP, a proton gradient uncoupler (Figure 4C). These results indicate that *yifK* is most likely an H^+^-coupled threonine symporter.

Based on phenotypic analysis of the *ΔbrnQ ΔlivKHMGF* double mutant B2289 strain, we assumed that serine is the second substrate for YifK. Indeed, an excess of serine almost completely inhibited the transport activity in the isogenic B2396 strain (with the wild-type *thrABC* operon) (Figure 4D). Next, we introduced the *ΔsdaC* mutation into B2396 and the triple mutant B2394 strains, thereby eliminating all known serine carriers, namely SstT, TdcC, and SdaC. When testing this pair of strains at 5 and 50 μM serine, we found that the transport activity of the quadruple mutant was negligible, whereas the strain with the wild-type *yifK* allele showed serine accumulation (Figure 4E). Notably, at 50 μM substrate, the rate of transport was even higher than that for threonine (compare Figure 3A and 4E). We determined the K_m_ and V_max_ of YifK for serine to be 89.4 ± 7.9 μM and 14.4 ± 0.6 nmol/min*mg DCW (average ± SD based on two biological replicates), respectively. Thus, YifK evidently exhibits double substrate specificity toward both threonine and serine.

Owing to the structural similarity between L-serine and L-homoserine, we also tested if YifK is capable of translocating the latter. To this end, we measured labeled threonine accumulation by the B2396 strain in the presence of an excess of unlabelled homoserine (Supplementary Figure S2). We did not detect significant inhibition of threonine transport. Thus, we conclude that YifK shows no specificity toward homoserine.

### 3.4. Involvement of LIV-I in serine uptake

**Figure 5:**
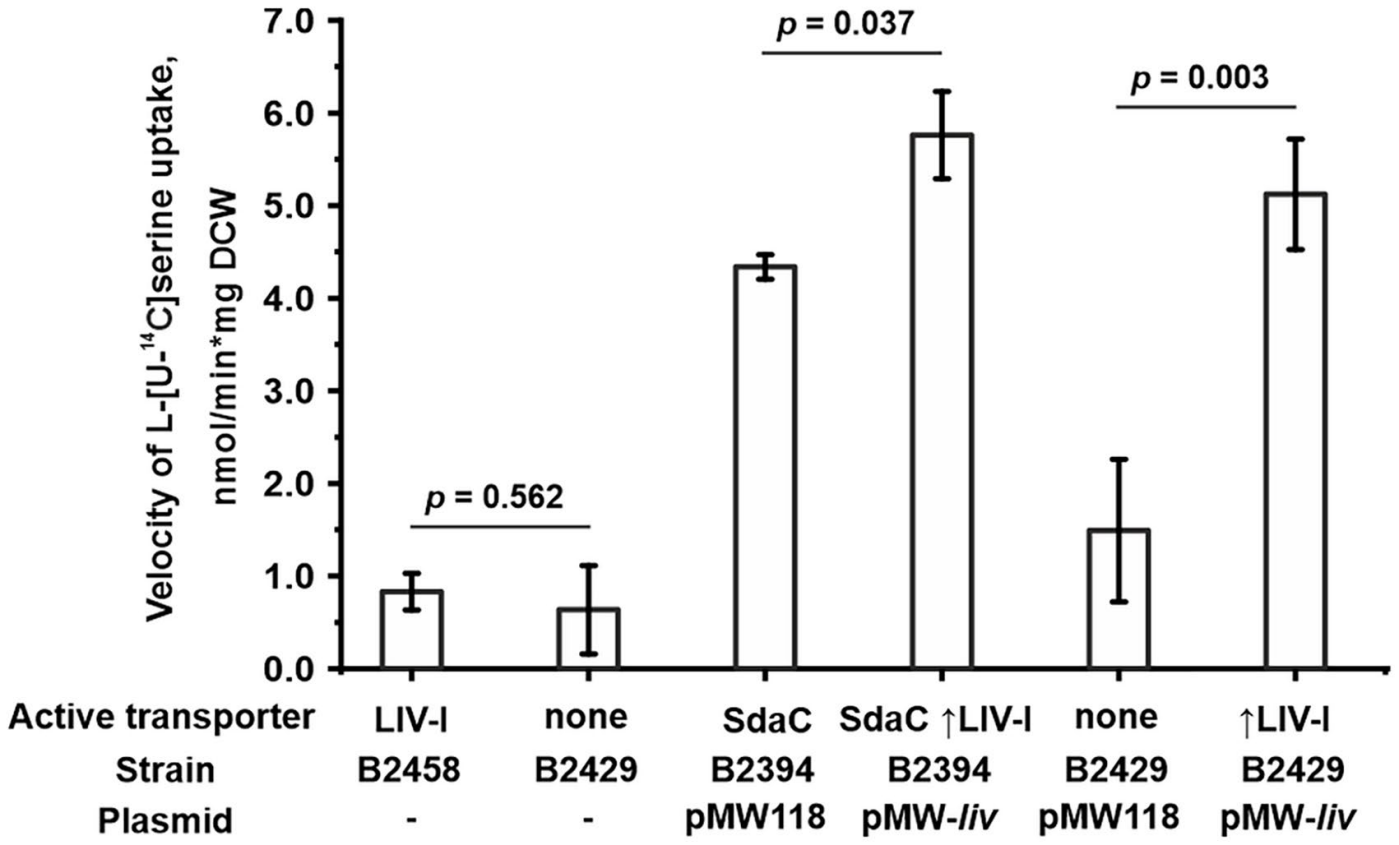
Involvement of the LIV-I carrier in L-serine uptake. The experiments were performed as described under “Transport assay” in the Materials and Methods. The velocity of uptake was measured at 100 μM L-serine. The reaction time was 1 min. The vertical arrow in “↑LIV-I” indicates that LIV-I is overexpressed in strains harboring the pMW-*liv* plasmid. The values shown are the average of three biological replicates; error bars indicate SD. The depicted *p-*values were calculated using the two-tailed Student’s *t-*test with unequal variances.

We then examined whether the LIV-I transport system participates in serine uptake along with the uptake of threonine, branched-chain amino acids, and phenylalanine. First, while performing phenotypic tests, we observed that an excess of serine did not inhibit the growth of the B1851 strain with LIV-I as the sole threonine uptake system (Figure 2B), thereby indicating that serine cannot compete with threonine for uptake *via* LIV-I. Next, we compared the B2429 and B2458 strains, of which the former carried deletions of the SstT, TdcC, LIV-I, YifK, and SdaC transport systems. The latter strain still possessed the LIV-I system. A comparison of the serine uptake rates between these two strains showed no significant differences (Figure 5). This result again indicates that LIV-I is unlikely to be involved in serine uptake. We then sought to improve the sensitivity of the assay by expressing the LIV-I locus from the pMW-*liv* plasmid. When comparing the serine transport activity of the B2394 strain possessing SdaC as the sole transporter transformed with either the pMW-*liv* plasmid or empty vector, we found only a minor increase in activity. Simultaneously, a comparison of the B2429 strain, which possessed no serine transport system, with the B2429 cells transformed with pMW-*liv* showed that LIV-I did exhibit some serine transport activity comparable to that of SdaC if the LIV-I locus was expressed from the plasmid rather than from a single chromosomal copy (Figure 5).

## 4. Discussion

Transport through the cytoplasmic membrane plays a pivotal role in maintaining the homeostasis of living cells and providing an adequate response to unstable environmental conditions. Despite extensive studies over the past few decades, our understanding of metabolite uptake and excretion is far from complete. For instance, 17 transport systems of *E. coli* cells have been characterized between 2013 and 2018 (Karp et al., 2018), and the process of exploring new carriers is still ongoing (Keseler et al., 2021).

In this study, we aimed to fill the gaps in our knowledge of the cellular threonine and serine transport subsystem. Starting from the report by Kruse et al. (2001), which suggested the existence of a putative serine-inhibitable threonine carrier, we identified YifK as a protein responsible for this transport activity *via* selection and analysis of spontaneous mutants. Our biochemical studies indicate that YifK is an H^+^-dependent transporter specific to both threonine and serine. Notably, the K_m_ value of YifK for threonine is 7.6-fold lower than that for serine. In contrast, V_max_ measured using serine as the substrate was 4.5 times higher compared with that obtained using threonine. These properties indicate that YifK is a bifunctional transporter whose primary substrate is either threonine or serine, depending on the concentration. Based on its higher specificity for threonine, we propose “*thrP”* as a new designation for the *yifK* gene.

Abolishing a substantial portion of threonine uptake *via* YifK inactivation allowed us to uncover the transporters responsible for the remaining activity. Using a strain carrying the *sstT*, *tdcC*, and *yifK* mutations, we found that *brnQ* is a multicopy suppressor of the threonine transport defect caused by inactivation of known transport systems. Phenotypic analysis of the mutants revealed that BrnQ clearly contributes to threonine transport at physiologically relevant substrate concentrations. However, *in vitro* uptake measurements showed that the activity of BrnQ was the lowest among the assessed transporters, possibly because of its relatively low specificity. In contrast, we found BrnQ to be responsible for threonine toxicity in a minimal medium containing a high concentration of threonine. Notably, selection of *brnQ*-mutant strains for threonine resistance has been reported recently in two studies (Rau et al., 2016; Radi et al., 2022). Our data provide, for the first time, convincing evidence regarding the direct involvement of BrnQ in threonine transport and define its place among other carriers constituting the uptake subsystem for this amino acid. Enforced by previous studies, our results indicate that BrnQ is a low-affinity but high-capacity carrier that becomes the main entry point when the substrate concentration reaches a toxic level. Thus, BrnQ has minor relevance for normal cellular physiology compared to YifK, but can be of great importance for biotechnological applications such as the construction of threonine producing strains.

Finally, once YifK and BrnQ activities were obviated, we could directly examine the threonine transport activity of the LIV-I system, which has been previously speculated to contribute to this process (Rahmanian et al., 1973; Robbins and Oxender, 1973; Templeton and Savageau, 1974). In our phenotypic assays, LIV-I provided nearly the same ability as YifK to consume exogenous threonine. However, *in vitro* measurement of threonine accumulation by the corresponding mutant strains (Figure 3A) showed a lower activity than that of YifK, thereby indicating that LIV-I is an auxiliary but still important transport system for threonine uptake. The apparent parity of the kinetic parameters of YifK and LIV-I (Figure 3B) can be likely explained by a distortion of the V_max_ value of LIV-I, as it was estimated using the strain possessing the LIV-I carrier in the overexpressed rather than in its native state. We also found that LIV-I had low-level serine uptake activity comparable to that of the dedicated SdaC carrier when the structural genes of LIV-I were overexpressed on the low-copy-number pMW118 plasmid. This result indicated that LIV-I is unlikely to be a serine carrier with significant physiological relevance.

**Figure 6:**
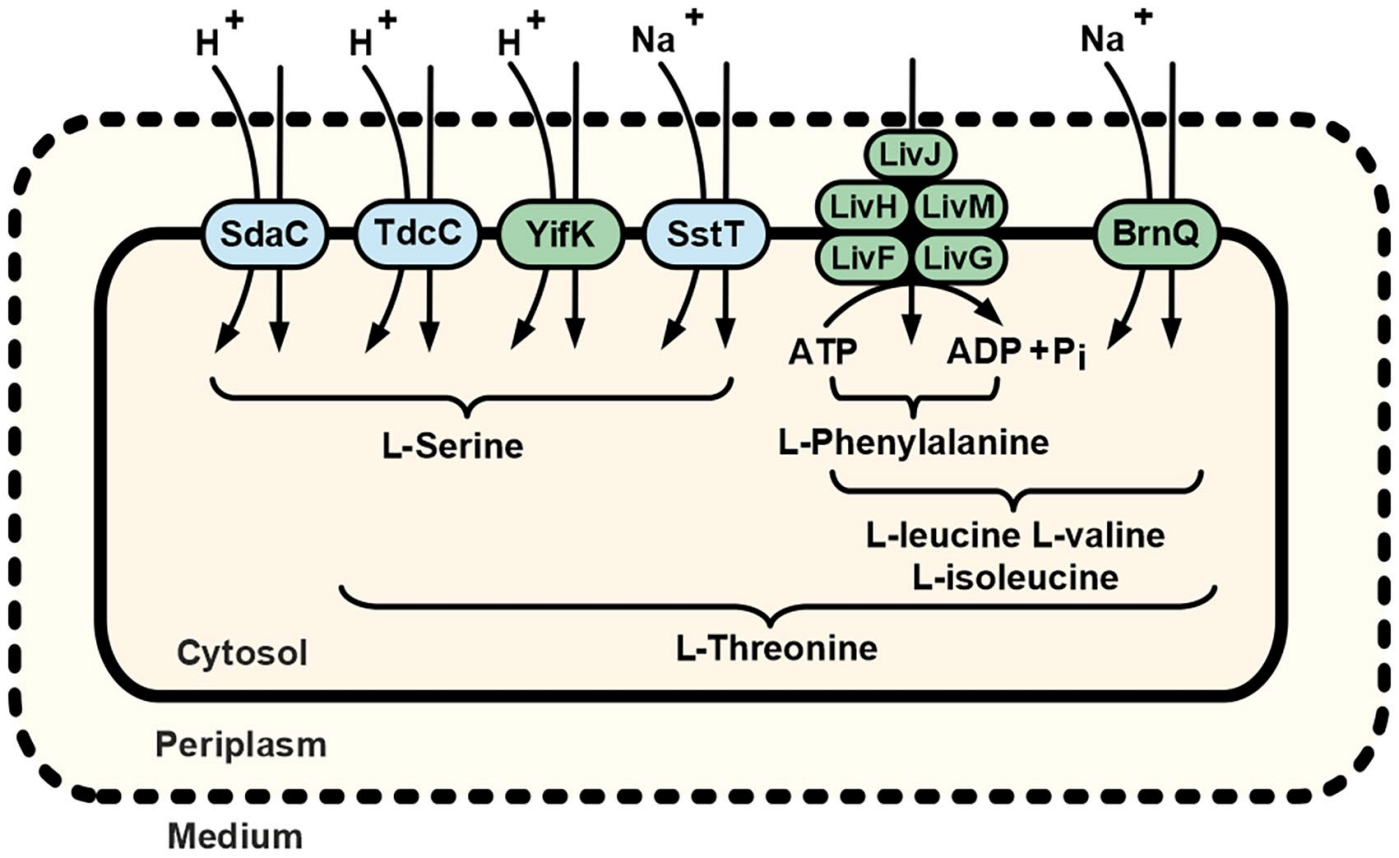
Schematic of the L-serine and L-threonine uptake routes in *E. coli* K-12 cells. Dotted and solid lines represent the outer and cytoplasmic membranes, respectively. Amino acid designations under braces indicate the substrate specificity of indicated transport systems. Green highlighting indicates the transport systems first discovered and reported in the present work (YifK) or characterized as carriers capable of translocating threonine (LIV-I and BrnQ). Previously known serine/threonine carriers (SdaC, TdcC, and SstT) are highlighted in blue. For more details, see the Discussion.

Importantly, cells of the B2394 strain (*∆sstT ∆tdcBCDE::neo ΔyifK ΔbrnQ ∆livKHMGF* mutant) exhibited negligible threonine uptake activity compared with that of the parental B2374 (*∆sstT ∆tdcBCDE::neo*). Similarly, the B2429 strain, which is isogenic to B2394 and additionally carries the *∆sdaC* mutation, has a drastically impaired ability to consume serine compared to that of either *the yifK^+^* or *sdaC^+^* strains. Taken together, these results suggest that all specific *E. coli* threonine and serine carriers have been identified. The residual uptake activity that became prominent at high concentrations of the substrates may be attributed to some proteins capable of translocating threonine and serine in an unspecific fashion and having little or no impact on their consumption from the medium under regular environmental conditions. Thus, we can summarize the already available and newly obtained data in a model of the cellular threonine and serine uptake subsystem. As depicted in Figure 6, threonine uptake activity is distributed among serine- and branched-chain amino acid-specific transport systems. The contribution hierarchy of each transporter under conditions of minimal medium and physiological substrate concentrations might be proposed as SstT = YifK > LIV-I > BrnQ. Although we did not evaluate SstT activity, it seemed to be roughly equal to that of YifK, as can be inferred from the results reported by Kruse et al. (2001). If the minor LIV-I-attributed serine transport activity is excluded from consideration, serine uptake proceeds through the single monospecific SdaC carrier, whereas the remaining activity is distributed among two bifunctional serine/threonine transporters: YifK, and SstT. In contrast to the listed carriers whose function is providing amino acids for anabolic reactions, the TdcC-attributed transport activity should rather be considered in relation to threonine and serine catabolism, controlled by the *tdcABCDEFG* operon, as TdcC is expressed exclusively under anaerobic conditions in a rich medium (Sumantran et al., 1990; Ogawa et al., 1997). Interestingly, threonine and serine uptake proceed *via* three distinct mechanisms, namely transport driven by ATP hydrolysis, H^+^-dependent symport, and Na^+^-dependent symport. This thereby ensures a flexible and reliable pathway for amino acid supply, regardless of the energetic state of the cell and the ion composition of a medium.

Although an overall complete picture of *E. coli* serine and threonine uptake systems has been obtained, there could still be some gaps in our understanding of serine/threonine transport. First, we examined the transport activity exclusively in minimal medium, which does not contain any amino acids. Such conditions are rare in the natural environment inhabited by *E. coli*. When cells grow in a complex medium containing a mixture of organic compounds, the exact contribution of each specific carrier may differ from that reported here. For instance, the LIV-I system is known to be tightly repressed in the presence of leucine (Rahmanian et al., 1973; Quay and Oxender, 1976), whereas TdcC is induced in a rich medium (Sumantran et al., 1990). Thus, we cannot rule out the existence of additional unidentified carriers expressed under specific conditions not examined in this study. Next, we faced a confusing discrepancy while measuring the transport activity of the transport mutants. We found a strain with active YifK but lacking LIV-I and BrnQ to accumulate threonine even faster than its counterpart, with all the carriers intact. This indicates a direct or indirect interplay between these transport systems. Further work is thus needed to test these hypotheses and to explore the response of the serine/threonine transport subsystem to specific environmental conditions.

## Data availability statement

The raw data supporting the conclusions of this article will be made available by the authors, without undue reservation.

## Author contributions

DB, TY, and TV: conceptualization. DB, TY, GA, and NA: methodology. AK, EP, EM, AS, and MK: investigation. DB, SS, and AN: project administration. SS: funding acquisition and supervision. DB, AK, and GA: writing the manuscript. All authors contributed to the article and approved the submitted version.

## Funding

This work was supported by Government Assignment No. AAAA-A20-120093090016-9 and a grant from the Ministry of Science and Higher Education of the Russian Federation No. 075–15–2019-1659.

## Conflict of interest

The authors declare that the research was conducted in the absence of any commercial or financial relationships that could be construed as a potential conflict of interest.

## Publisher’s note

All claims expressed in this article are solely those of the authors and do not necessarily represent those of their affiliated organizations, or those of the publisher, the editors and the reviewers. Any product that may be evaluated in this article, or claim that may be made by its manufacturer, is not guaranteed or endorsed by the publisher.
