# Supplementary material for: A study on L-threonine and L-serine uptake in Escherichia coli K-12
Source: Front Microbiol. 2023 Mar 21;14:1151716. doi: 10.3389/fmicb.2023.1151716 (PMC10070963; doi:10.3389/fmicb.2023.1151716)
Supplement: Supplementary file 1 [file Data_Sheet_1.PDF]

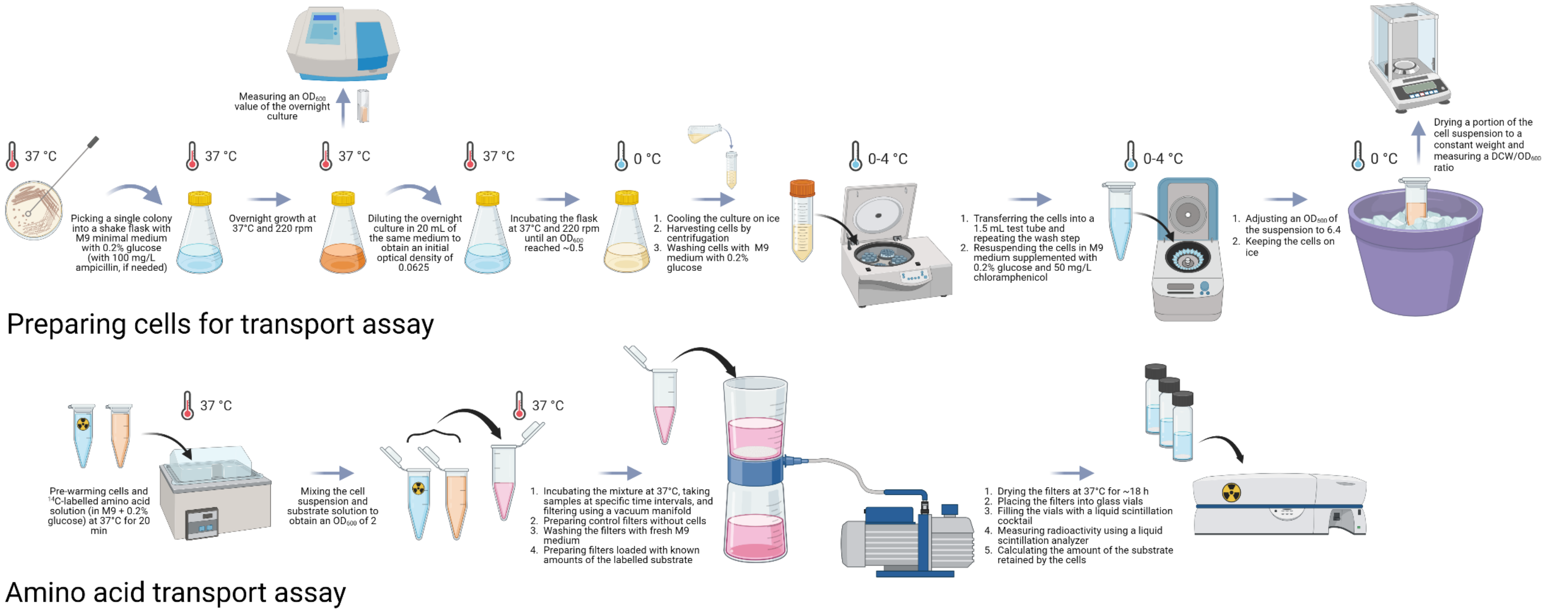

Supplementary Figure 1. General outline of the amino acid transport experiments. A detailed description is presented under “Transport assay” in the Materials and Methods. Created with BioRender.com.

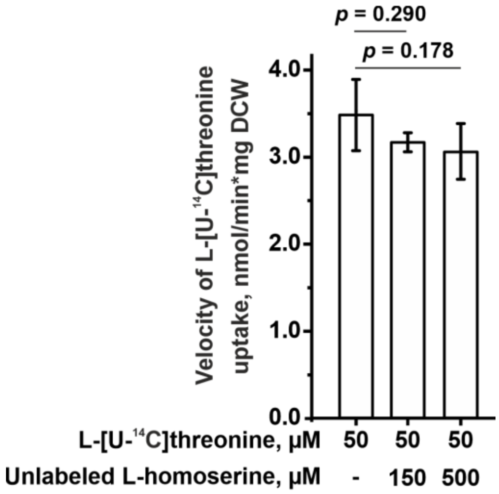

Supplementary Figure 2. The effect of excessive unlabeled L-homoserine on the L-threonine transport activity of YifK. The experiments were performed using cells of the B2396 strain as described under “Transport assay” in the Materials and Methods. The reaction time was 1 min. The values shown are the average of three biological replicates; error bars indicate SD. The depicted *p*-values were calculated using the two-tailed Student’s *t*-test with unequal variances.
